# The clinical efficacy of modified xiaoyaosan in the treatment of liver Qi stagnation-type chloasma from the starting point of liver

**DOI:** 10.1097/MD.0000000000045517

**Published:** 2025-10-31

**Authors:** Ke-Dang Ma, Qing-Qiang Xu, Xiang-Lan Wang, Zhang-Jun Li

**Affiliations:** a Department of Dermatology, Shaanxi Provincial Hospital of Chinese Medicine, Xi’an City, Shaanxi Province, China; b Department of Dermatology, Second Affiliated Hospital of Xi’an Jiaotong University, Xi’an City, Shaanxi Province, China.

**Keywords:** chloasma, liver qi stagnation, Modified Xiaoyaosan, treatment efficacy

## Abstract

Chloasma (melasma) is a common pigmentary disorder characterized by hyperpigmented facial macules with a high recurrence rate. In Traditional Chinese Medicine, liver qi stagnation is considered a key pathogenesis. This study aimed to evaluate the clinical efficacy and safety of Modified Xiaoyaosan, a classical liver-soothing herbal formula, as an adjunct to conventional therapy in patients with liver qi stagnation-type chloasma. A retrospective analysis was conducted on 78 female patients diagnosed with liver qi stagnation-type chloasma who received treatment at our hospital. Based on their treatment regimens, patients were categorized into a control group (treated with vitamin C, vitamin E, and topical tranexamic acid) and a study group (treated with the same regimen plus Modified Xiaoyaosan) for a duration of 12 weeks. Clinical efficacy, skin lesion scores, VISIA skin imaging parameters, quality of life (assessed using the Dermatology Life Quality Index), serum oxidative stress markers (including superoxide dismutase and malondialdehyde), recurrence rate, and adverse events were retrospectively evaluated and compared between the 2 groups. The study group showed significantly higher total clinical efficacy (94.87% vs 79.49%, *P* < .05), greater improvement in skin lesion scores and VISIA parameters (*P* < .001), and lower Dermatology Life Quality Index scores at 4 and 12 weeks (*P* < .001). Serum superoxide dismutase levels increased while malondialdehyde levels decreased more significantly in the study group (*P* < .001). Recurrence rate was lower in the study group (2.56% vs 17.95%, *P* < .05), with no significant difference in adverse event rates. Modified Xiaoyaosan combined with conventional Western treatment effectively improves clinical symptoms, reduces oxidative stress, enhances skin quality and quality of life, and lowers recurrence in liver qi stagnation-type chloasma, with good safety and tolerability.

## 1. Introduction

Chloasma is a prevalent dermatological disease, characterized predominantly by facial hyperpigmentation. The lesions typically present in a symmetrical distribution, varying in size and irregular in shape, with patches merging into larger areas. The borders are well-defined, the surface is smooth and level with the skin, and there are no scales or inflammatory responses.^[1,2]^ Although both men and women may develop chloasma, it is more frequent in females, particularly those of childbearing age or postpartum. The hyperpigmentation tends to lighten slightly in winter and deepen in summer.^[3]^ The etiology of chloasma is complicated, often resulting from a combination of internal and external causes that disrupt the skin barrier and trigger metabolic abnormalities. Traditional Chinese medicine (TCM) associates the occurrence of chloasma with the liver, spleen, and kidneys, with liver qi stagnation being a primary factor. In contrast, Western medicine attributes the condition to genetics, endocrine abnormalities, ultraviolet irradiation, and changes in the body’s oxygen radical content. Histological alterations are monitored in the epidermis, basement membrane, and upper dermis during the disease’s progression.^[4,5]^ Due to its chronic nature and irregular response, chloasma remains challenging to treat, with no definitive cure currently available.

With the rising standards of living and increasing aesthetic demands, there is a growing focus on treating cosmetically disfiguring conditions like chloasma. There is an urgent need for safe and effective treatment modalities. Common Western medical treatments for chloasma include vitamin C, tranexamic acid, glutathione, and tea polyphenols,^[6–8]^ or topical treatments with decoloring agents and laser face beautifiers. While these methods can be effective, they may also disrupt the skin barrier and cause inflammatory side effects.^[9]^ At present, there exist numerous methods for treating chloasma in clinical practice. Compared to Western medical treatments, TCM holds certain advantages. Techniques such as acupuncturing, cupping, facial massage, and the application of TCM masks can comprehensively enhance qi-blood circulation, improve facial blood circulation, and boost skin metabolism. These methods also combat and eliminate oxygen radicals, thereby achieving the goal of lightening and removing chloasma.^[10,11]^ Research denotes that chloasma bleaching pertains to surface thickening. Microneedles can complement local treatments by ameliorating skin photoaging, while oral tranexamic acid can supplement local treatments by suppressing stem cell factors. During treatment, it is crucial to avoid damaging the skin barrier and stimulating angiogenesis.^[12]^

In this study, we adopted a combination of both TCM and Western medicine to treat patients with liver qi stagnation-type chloasma admitted to our hospital. Alongside the standard oral administration of vitamin C, and vitamin E, and topical application of tranexamic acid, we incorporated Modified Xiaoyaosan as an adjunct therapy. We evaluated the clinical efficacy and safety of this integrative treatment approach, aiming to provide additional insights into the clinical management of chloasma.

## 2. Clinical data

### 2.1. Inclusion and exclusion criteria

Inclusion criteria: Patients must meet the diagnostic criteria for chloasma established by the Pigment Epidemiology Group of the Dermatological Disease Committee of the Chinese Association of Integrative Medicine.^[13]^ Facial patches range from light brown to dark brown in terms of color, with clear boundaries and often symmetrical distribution. There is no inflammation or scales, and no remarkable subjective symptoms are present. The condition exhibits a seasonal nature, with more severe symptoms in summer and milder symptoms in winter. The TCM differentiation type is identified as liver qi stagnation syndrome, characterized by light brown or brown patches on the face, accompanied by dysmenorrhea or irregular menstruation, distending pain in hypochondrium, anxiety and irritability or depression, a pale red tongue, a white and thin coating of the tongue, as well as weak and slow pulse. The course of disease is more than 1 month. Patients are exclusively female. Age ranges from 18 to 65 years old. Complete clinical data and good compliance are required.

Exclusion criteria: Patients with hematological disorders or abnormal renal and liver function; patients who have received treatments affecting pigment metabolism within the past month; patients with sunlight allergy; patients who are allergic to the experimental drugs used in this study; patients with nevus fuscocaeruleus zygomaticus, lichen planus pigmentosus, or other conditions causing hyperpigmentation; pregnant or lactating women; Patients with mental or cognitive disorders; and patients with incomplete clinical data or unable to cooperate with the study requirements.

### 2.2. General data

A total of 78 patients diagnosed with liver qi stagnation-type chloasma and treated at our hospital were retrospectively enrolled in this study. Based on the treatment modalities they received, patients were categorized into the control group (n = 39) and the study group (n = 39). There were no statistically significant differences between the 2 groups with respect to baseline characteristics such as age, disease duration, and lesion distribution (*P* > .05), indicating good comparability (see Table 1). This retrospective study was approved by the Ethics Committee of our hospital.

**Table 1:** Comparison of general data between the 2 groups (x̄ ± s, n).

Group	n	Age (yr)	Course of disease (yr)	Location of patches		
				Cheekbone	Forehead	Generalized
Study group	39	41.95 ± 5.33	5.25 ± 0.42	14	10	15
Control group	39	42.03 ± 5.92	5.30 ± 0.39	13	8	18
*χ^2^*/*t*		0.063	0.548	0.532		
*P*		.950	.588	.766		

### 2.3. Treatment methods

In the control group, patients were orally administered 100 mg vitamin C tablets 3 times daily (manufactured by Northeast Pharmaceutical Group Shenyang First Pharmaceutical Co., Ltd.), and 100 mg vitamin E soft capsules once daily (manufactured by Zhejiang Medicine Co., Ltd.). Additionally, 0.5% tranexamic acid injection was taken to wet 2 layers of gauze applied to the chloasma lesions for 20 minutes, once daily (manufactured by Changchun Tiancheng Pharmaceutical Co., Ltd.).

As for the study group, in addition to the treatments employed within the control group, patients were orally administered a Chinese herbal decoction Modified Xiaoyaosan, prepared by our hospital’s pharmacy using a drug-decocting machine. The specifications were 1 dose of herbal medicine prepared into 2 bags, each bag containing 100 ml. The decoction was taken once daily, 30 minutes after breakfast and dinner. The basic prescription is as follows: 12 g *Bupleuri Radix* (Chaihu in Chinese), 12 g *Angelicae Sinensis Radix* (Danggui), 6 g *Zingiberis Rhizoma Recens* (Shengjiang), 12 g *Paeoniae Radix Alba* (Baishao), 15 g *Poria* (Fuling), 12 g *Atractylodis Macrocephalae Rhizoma* (Baizhu), 6 g *Menthae Haplocalycis Herba* (Bohe), and 6 g *Glycyrrhizae Radix et Rhizoma* (Gancao). According to the patient’s actual situation and the synthesis of the 4 diagnostic methods (observation, auscultation and olfaction, inquiry, pulse feeling and palpation), the prescription was adjusted. For patients with constipation, additional herbs such as *Rhei Radix et Rhizoma* (Dahuang) and *Cannabis Fructus* (Huomaren) were added. For patients with a bitter taste and dry throat, herbs such as *Moutan Cortex* (Mudanpi), *Gardeniae Fructus* (Zhizi), and *Scutellariae Radix* (Huangqin) were given. For patients with irregular menstruation, herbs such as *Ligustri Lucidi Fructus* (Nüzhenzi) and *Cyperi Rhizoma* (Xiangfu) were added. reevaluation was conducted every 2 weeks, and the decoction was modified as per the patient’s condition and alterations in the disease status.

### 2.4. Evaluation of efficacy

As instructed by previous literature,^[14]^ we assessed the clinical efficacy after 12 weeks of treatment in both groups: Recovery: Reduction in the area of hyperpigmentation ≥ 90%, skin color basically normal, total lesion score decreased by ≥ 80%; Marked effectiveness: Reduction in the area of hyperpigmentation by 60% to 89%, conspicuous lightening of hyperpigmentation color, total lesion score decreased by 50% to 79%; Effectiveness: Reduction in the area of hyperpigmentation by 30% to 59%, slight lightening of the color, total lesion score decreased by 30% to 49%; No effectiveness: Reduction in the area of hyperpigmentation < 30% or increase, no significant change or darkening in hyperpigmentation color, total lesion score decreased by < 30%. Overall effective rate = (recovery + marked effect + effective) cases/ total cases × 100%.

### 2.5. Skin lesion scores

The skin lesions of both cohorts were contrasted before and after 12 weeks of treatment, including lesion area, color, and total scores. Lesion area scores: 0 points: No lesions; 1 point: Lesion area < 2 cm², mild lesions; 2 points: Lesion area 2 to 4 cm², moderate lesions; 3 points: Lesion area > 4 cm², severe lesions. Lesion color scores: Normal skin color scores 0 points, light brown scores 1 point, brown scores 2 points, dark brown scores 3 points. Total lesion score = lesion area score + lesion color score.

### 2.6. Skin-related parameters

The VISIA skin imaging analyzer was exploited to evaluate the pigmented spots, wrinkles, texture, pores, and porphyrin deposition of the 3 groups of patients preceding and following 12 weeks of treatment. Scores were converted to a percentage scale, where lower scores signify better skin recovery.

### 2.7. Quality of life

We compared the quality of life of the 2 cohorts of patients prior to treatment, at 4 weeks, and at 12 weeks of treatment. The Dermatology Life Quality Index (DLQI)^[15]^ was adopted for assessment, encompassing symptoms and physical sensation, psychological sensation, outdoor activities, clothing choices, social and recreational activities, sports and exercises, work, relationships with family or friends, sexual life, and daily life, with a total score ranging from 0 to 30 points. DLQI scores of 0 to 1 indicate no impact on the patient’s life; scores of 2 to 5 denote a minor impact; scores of 6 to 10 reflect a moderate impact; scores of 11 to 20 suggest a very large impact; and scores of 21 to 30 demonstrate an extremely large impact on the patient’s life.

### 2.8. Oxidative stress indicators

Peripheral venous blood samples were harvested from the 2 groups before treatment, at 4 weeks, and at 12 weeks of treatment. Following centrifugation, the serum contents of superoxide dismutase (SOD) and malondialdehyde (MDA) were gauged. SOD was detected using the xanthine oxidase method, and MDA was measured via the thiobarbituric acid colorimetric method, strictly following the instructions provided with the assay kits.

### 2.9. Recurrence and adverse reaction rates

Subsequent to 12 weeks of treatment, patients were followed up to record whether there was any recurrence during this period. The recurrence rate was calculated as the number of recurrence cases divided by the total number of cases, multiplied by 100%. Simultaneously, adverse reactions such as gastrointestinal reactions and drug allergies were observed in both cohorts of patients during treatment. The adverse reaction rate was calculated as the number of patients with adverse reactions divided by the total number of patients, multiplied by 100%.

### 2.10. Statistical analysis

SPSS 22.0 software was introduced to process and analyze all data in this research. Measurement data were displayed as (‾x ± s), and inter-group comparisons were made through *t*-tests. Enumeration data were presented as percentages, and comparisons were made through the *χ*^2^ test. A *P* value <.05 was considered statistically significant.

## 3. Results

### 3.1. Clinical efficacy in the 2 groups

Following 12 weeks of treatment with different regimens, the numbers of patients within the study group who achieved recovery, marked effectiveness, effectiveness, and no effectiveness were 16, 15, 6, and 2, respectively. The total effective rate in the study cohort reached 94.87%, markedly higher than the 79.49% within the control group, with a statistically significant disparity (*P* < .05), as exhibited in Table 2. Our findings confirmed that the adjunctive application of Modified Xiaoyaosan was effective for chloasma of liver-qi stagnation type, and the combined treatment of TCM and Western medicine was more effective than Western medicine alone.

**Table 2:** Comparison of clinical efficacy between the 2 groups (n, %).

Group	n	Recovery	Marked effectiveness	Effectiveness	No effectiveness	Total effectiveness rate
Study group	39	16 (41.03)	15 (38.46)	6 (15.38)	2 (5.13)	37 (94.87)
Control group	39	9 (23.08)	14 (35.90)	8 (20.51)	8 (20.51)	31 (79.49)
*χ^2^*						4.129
*P*						.042

### 3.2. Skin lesion scores before and after treatment in the 2 groups

Prior to treatment, there was no substantial statistical difference in the lesion area, lesion color, and total score between the 2 cohorts (*P* > .05). Following 12 weeks of treatment, the lesion area in the study cohort was (1.03 ± 0.12) points, the lesion color was (0.97 ± 0.11) points, and the total score was (2.01 ± 0.23) points, all of which were lower than those within the control group during the same period, with statistically significant variances (*P* < .05), as displayed in Table 3. From these results, it could be concluded that compared with the treatment using only vitamin C, and vitamin E, and the topical application of tranexamic acid in Western medicine, the combined treatment with TCM decoction Modified Xiaoyaosan provided better improvement in skin lesions for patients.

**Table 3:** Comparison of skin lesions before and after treatment between the 2 groups (x̄ ± s, points).

Group	n	Lesion area		Lesion color		Total score	
		Before treatment	After treatment	Before treatment	After treatment	Before treatment	After treatment
Study group	39	2.15 ± 0.21	1.03 ± 0.12*	2.08 ± 0.31	0.97 ± 0.11*	4.22 ± 0.45	2.01 ± 0.23*
Control group	39	2.19 ± 0.25	1.45 ± 0.16*	2.12 ± 0.26	1.29 ± 0.15*	4.30 ± 0.41	2.76 ± 0.29*
*t*		0.765	13.114	0.617	10.743	0.821	12.654
*P*		.447	<.001	.539	<.001	.414	<.001

* Compared with before treatment, *P* < .05.

### 3.3. Results of skin image analysis before and after treatment in the 2 groups

Prior to treatment, there was no significantly statistical difference in the scores for pigmented spots, wrinkles, texture, pores, and porphyrins between the 2 cohorts (*P* > .05). Subsequent to treatment, scores for all items in both groups showed substantial improvement compared to before treatment, with greater improvement observed in the study group. Specifically, the study cohort exhibited scores of (21.72 ± 3.08) for pigmented spots, (21.13 ± 2.33) for wrinkles, (22.46 ± 2.38) for texture, (27.66 ± 3.88) for pores, and (22.03 ± 2.35) for porphyrins, all lower than those within the control group during the same period (*P* < .05), as displayed in Table 4. Our findings demonstrated that Modified Xiaoyaosan as an adjuvant therapy for chloasma of liver qi stagnation type dramatically improved skin quality, abating pigmented spots, wrinkles, texture, pores, and porphyrins.

**Table 4:** Comparison of skin image analysis outcomes between the 2 groups before and after treatment (x̄ ± s, points).

Group	n	Pigmented spots		Wrinkles		Texture		Pores		Porphyrins	
		Before treatment	After treatment	Before treatment	After treatment	Before treatment	After treatment	Before treatment	After treatment	Before treatment	After treatment
Study group	39	45.13 ± 4.66	21.72 ± 3.08*	43.05 ± 4.26	21.13 ± 2.33*	47.97 ± 5.14	22.46 ± 2.38*	63.35 ± 6.92	27.66 ± 3.88*	40.79 ± 3.79	22.03 ± 2.35*
Control group	39	45.26 ± 5.12	37.61 ± 3.40*	42.94 ± 4.99	33.75 ± 3.16*	47.41 ± 4.55	36.71 ± 3.19*	64.02 ± 6.75	41.08 ± 4.61*	41.12 ± 4.22	35.72 ± 3.21*
*t*		0.117	21.631	0.105	20.074	0.509	22.360	0.433	13.909	0.363	21.490
*P*		.907	<.001	.917	<.001	.612	<.001	.666	<.001	.717	<.001

* Compared with the levels before treatment, *P* < .05.

### 3.4. Quality of life scores in both groups preceding and following treatment

DLQI scores are an effective indicator reflecting the quality of life of patients with dermatological diseases. Prior to the therapy, no notable statistical variance was discovered in DLQI scores between the 2 cohorts (*P* > .05). At 4 and 12 weeks post-treatment, both cohorts displayed a substantial decrease in quality of life scores compared to before treatment. At 4 weeks after treatment, the study cohort had a DLQI score of (21.35 ± 2.33), and at 12 weeks following treatment, the study cohort had a DLQI score of (17.88 ± 1.87), both of which were lower than those within the control group during the same period (*P* < .05). Refer to Table 5. This confirmed that vis-à-vis treatment with Western medicine alone, the addition of Modified Xiaoyaosan as adjuvant therapy for liver qi stagnation-type chloasma better improved the quality of life of patients following treatment.

**Table 5:** Comparison of DLQI scores between the 2 groups preceding and following treatment (x̄ ± s, points).

Group	n	Pretreatment	4 wk of treatment	12 wk of treatment
Study group	39	26.87 ± 2.43	21.35 ± 2.33*	17.88 ± 1.87*
Control group	39	27.71 ± 2.64	25.78 ± 1.49*	20.62 ± 1.95*
*t*		1.462	11.280	6.333
*P*		.148	<.001	<.001

* Compared with the levels before treatment, *P* < .05.

### 3.5. Oxidative stress indicators in the 2 groups before and after treatment

Preceding treatment, no statistical significance was confirmed in the variances between the 2 groups’ SOD and MDA levels (*P* > .05). Following 12 weeks of treatment, both cohorts displayed improvement compared to pretreatment levels, with the study group showing more remarkable improvement. At 12 weeks of treatment, the SOD level in the study cohort stood at (168.73 ± 15.43) ng/mL, and the MDA level attained (4.12 ± 0.36) nmol/mL. The SOD level was heightened in the study group as opposed to the control group during the same period, whereas the MDA level was lowered, with statistically significant variances (*P* < .05), as exhibited in Table 6. This corroborated that, versus conventional Western medicine treatment alone, the addition of Modified Xiaoyaosan as adjuvant therapy better improved oxidative stress parameters in patients with chloasma, ameliorating oxidative damage in patients.

**Table 6:** Comparison of SOD and MDA levels between the 2 groups pre- and post-treatment (x̄ ± s).

Group	n	SOD (ng/mL)		MDA (nmol/mL)	
		Pretreatment	12 wk of treatment	Pretreatment	12 wk of treatment
Study group	39	100.45 ± 12.33	168.73 ± 15.43*	5.34 ± 0.61	4.12 ± 0.36*
Control group	39	101.84 ± 11.65	135.94 ± 14.91*	5.39 ± 0.64	4.67 ± 0.49*
*t*		0.512	9.544	0.353	5.649
*P*		.610	<.001	.725	<.001

* Compared with the levels before treatment, *P* < .05.

### 3.6. Adverse reaction and recurrence rates in the 2 groups

Dryness, skin desquamation, itching, mild tingling, and minor gastrointestinal reactions are prevalent adverse reactions in the integrated TCM and Western medicine treatment of chloasma. The total incidence of adverse reactions within the study group attained 20.51%, which was higher than the control group’s 12.82%, but the variance was not considered statistically significant (*P* > .05). Chloasma tends to recur subsequent to treatment. In our experiment, only 1 case relapsed during the follow-up period within the study group, with a recurrence rate of 2.56%, much lower than the control group’s 17.95% (7/39), which contained statistical significance (*P* < .05), as shown in Table 7. Our discoveries demonstrated that Modified Xiaoyaosan as adjuvant therapy for liver qi stagnation-type chloasma effectively controlled adverse reactions and post-treatment recurrence.

**Table 7:** Comparison of adverse reaction and recurrence rates between the 2 groups (n, %).

Group	n	Dryness	Skin desquamation	Itching	Mild tingling	Gastrointestinal reactions	Adverse reaction rate	Recurrence rate
Study group	39	1 (2.56)	3 (7.69)	1 (2.56)	2 (5.13)	1 (2.56)	8 (20.51)	1 (2.56)
Control group	39	1 (2.56)	2 (5.13)	1 (2.56)	1 (2.56)	0 (0)	5 (12.82)	7 (17.95)
*χ^2^*							0.831	5.014
*P*							.362	.025

## 4. Discussion

With the development of the economy and changes in people’s work and lifestyle, more and more individuals are experiencing significant stress in their work and daily lives. This has led to the adoption of unhealthy habits such as staying up late, prolonged exposure to electronic products like computers and smartphones, lack of exercise, and outdoor activities, causing a continuous rise in the proportion of sub-health.^[16]^ Under these conditions, the incidence of chloasma has also been increasing year by year. The occurrence of chloasma is not only related to factors such as ultraviolet irradiation and changes in hormone levels but also to pregnancy, hormonotherapy, and long-term exposure to sunlight.^[17,18]^ As a difficult-to-treat cosmetically disfiguring condition, although chloasma does not endanger the patient’s life, it can affect the patient’s appearance and cause considerable distress. Patients often experience augmented mental stress, which impacts their quality of life.^[19,20]^

Chloasma is presently a challenging problem in the medical field. It not only has a long course and is prone to recurrence but is also difficult to cure. Modern medicine has not fully understood the pathogenic factors of chloasma, but it is extensively recognized that genetics, endocrine disorders, ultraviolet light, cosmetics, and emotional factors all play inducing parts in its pathogenesis. Additionally, the onset of chloasma is also associated with some chronic diseases such as polycystic ovarian syndrome, hepatitis, cirrhosis, tuberculosis, and menstrual disorders.^[21–23]^ Mahjour et al^[24]^ have discovered that for chloasma triggered by oligomenorrhea, besides improving hypomenorrhea, systemic anti-estrogenic therapy can also dampen melanin production by attenuating the process of α-melanocytes stimulating hormones, hence ameliorating chloasma. Systemic treatment is more efficacious than local treatment for chloasma. Conventional Western medical treatment approaches have significant limitations. TCM, as a unique medical system, addresses chloasma through dialectical therapy, which has unique advantages. In the middle of treatment, it comprehensively regulates patients’ conditions based on a holistic concept tailored to their constitutions, modifies prescriptions flexibly according to the condition, and adapts treatment accordingly.^[25]^

Liver qi stagnation type is a relatively basic and common syndrome in chloasma. Patients with this syndrome are more prevalent, making them an important subject for our clinical study. In this study, we adopted Modified Xiaoyaosan to assist in the treatment of liver qi stagnation-type chloasma patients. The findings suggested that vis-à-vis the control cohort treated only with conventional vitamin C, E, and tranexamic acid, the study cohort displayed higher clinical efficacy, lower recurrence rate, better improvement in skin lesions including pigmented spots, wrinkles, texture, pores, and porphyrins, lower DLQI scores, higher SOD levels, as well as lower MDA levels. Our experimental outcomes denoted that compared to treatment with only vitamin C, E, and tranexamic acid, adding Modified Xiaoyaosan as an adjuvant therapy dramatically enhanced the clinical effects of treating chloasma, substantially enhancing skin condition, abating oxidative stress damage, lowering recurrence rates, and demonstrating good safety. Our findings are aligned with previous reports. Tang et al^[26]^ have discovered that oral herbal medicine combined with conventional therapy outperforms conventional therapy alone, notably lowering skin damage scores, with no notable difference in adverse reaction rates between the 2 treatments. Su et al^[27]^ have also found that versus the control group treated with tranexamic acid, the experimental group treated with a self-designed spot-eliminating and blood circulation-promoting formula shows a notably higher total effective rate, lowered severity index scores for chloasma area, and a lower incidence of adverse reactions, possibly due to the inhibition of serum sex hormone expressions and tyrosinase absorbance. The achievement of our study is attributed to the formulation of Modified Xiaoyaosan, which consists of *Bupleuri Radix* (Chaihu), *Angelicae Sinensis Radix* (Danggui), *Zingiberis Rhizoma Recens* (Shengjiang), *Paeoniae Radix Alba* (Baishao), *Poria* (Fuling), *Atractylodis Macrocephalae Rhizoma* (Baizhu), *Menthae Haplocalycis Herba* (Bohe), and *Glycyrrhizae Radix et Rhizoma* (Gancao), where Chaihu acts as the sovereign drug, Baishao and Danggui as minister drugs, Shengjiang, Baizhu, and Bohe as assistant drugs, and Gancao as the envoy drug. These herbs work together to nourish and regulate the liver and spleen, modulate qi and remove blood stasis, as well as activate blood and clear heat, fundamentally alleviating liver stress^[28]^ and treating chloasma. Modern pharmacology recognizes that the primary active ingredients in Chaihu, *Saikosaponins*, boast anti-inflammatory, adrenal-stimulating, immune-enhancing, liver-protecting, and protein synthesis-promoting effects.^[29]^ Chaihu, Danggui, Baishao, Shengjiang, and Fuling all exert good antioxidant effects, whereas Danggui and Baizhu are rich in amino acids and vitamin A, which help nourish the skin. Xiaoyaosan works best for the early treatment of chloasma through various means such as relieving negative emotions, antioxidation, and hormone regulation.^[30]^ As opposed to conventional Western medicine alone, it is more effective and does not increase the incidence of adverse events, dramatically improving the quality of life of patients following treatment.

Nevertheless, several limitations should be acknowledged. First, dermoscopic and histopathological evaluations were not included in this retrospective analysis due to the lack of relevant data in patient records. Given the cosmetic nature of facial chloasma and the noninvasive treatment approach adopted in this study, routine skin biopsies were not performed to avoid additional trauma or patient discomfort. Additionally, dermoscopic imaging was not systematically applied during the clinical evaluation. Although these objective assessments could have offered more detailed insights into pigmentation patterns and histological alterations before and after treatment, the current study employed a comprehensive set of outcome measures, including standardized clinical efficacy criteria, VISIA skin imaging, biochemical oxidative stress markers, and dermatology-specific quality of life indices, which together provide robust and multidimensional evidence supporting the effectiveness of Modified Xiaoyaosan as an adjunct therapy. Future prospective studies incorporating dermoscopic and histopathological endpoints are warranted to further elucidate the underlying mechanisms and skin structural changes associated with therapeutic response.

In summation, the adjuvant therapy of Modified Xiaoyaosan for liver qi stagnation-type chloasma displays conspicuous clinical efficacy. It helps ameliorate skin damage, abate oxidative stress damage, attenuate recurrence rates, and boost patient quality of life. Moreover, it boasts a lower incidence of adverse reactions. This treatment approach is safe and reliable for liver qi stagnation-type chloasma and deserves widespread clinical application.

## Acknowledgments

Thanks to the support of the Foundation and the contributions of all the participants.

## Author contributions

**Conceptualization:** Ke-Dang Ma.

**Data curation:** Ke-Dang Ma, Qing-Qiang Xu, Xiang-Lan Wang.

**Formal analysis:** Ke-Dang Ma, Xiang-Lan Wang.

**Investigation:** Ke-Dang Ma, Qing-Qiang Xu.

**Methodology:** Ke-Dang Ma, Qing-Qiang Xu, Zhang-Jun Li.

**Resources:** Ke-Dang Ma, Qing-Qiang Xu, Xiang-Lan Wang.

**Software:** Qing-Qiang Xu, Xiang-Lan Wang.

**Supervision:** Zhang-Jun Li.

**Writing – original draft:** Ke-Dang Ma.

**Writing – review & editing:** Zhang-Jun Li.
